# Stochastic SIRC epidemic model with time-delay for COVID-19

**DOI:** 10.1186/s13662-020-02964-8

**Published:** 2020-09-18

**Authors:** F. A. Rihan, H. J. Alsakaji, C. Rajivganthi

**Affiliations:** 1 Department of Mathematical Sciences, College of Science, United Arab Emirates University, Al-Ain, 15551 UAE; 2 School of Applied Mathematics, Getulio Vargas Foundation, Rio de Janeiro, RJ 22250-900 Brazil

**Keywords:** Brownian motion, COVID-19, Cross-immunity, Extinction, Stationary distribution, Stochastic SIRC model

## Abstract

Environmental factors, such as humidity, precipitation, and temperature, have significant impacts on the spread of the new strain coronavirus COVID-19 to humans. In this paper, we use a stochastic epidemic SIRC model, with cross-immune class and time-delay in transmission terms, for the spread of COVID-19. We analyze the model and prove the existence and uniqueness of positive global solution. We deduce the basic reproduction number ${\mathcal{R}}_{0}^{s}$ for the stochastic model which is smaller than ${\mathcal{R}}_{0}$ of the corresponding deterministic model. Sufficient conditions that guarantee the existence of a unique ergodic stationary distribution, using the stochastic Lyapunov function, and conditions for the extinction of the disease are obtained. Our findings show that white noise plays an important part in controlling the spread of the disease; When the white noise is relatively large, the infectious diseases will become extinct; Re-infection and periodic outbreaks can occur due to the existence of feedback time-delay (or memory) in the transmission terms.

## Introduction

The ongoing pandemic coronavirus disease (COVID-19) has become a worldwide emergency. This infectious disease is spreading fast, endangering a large number of people’s health, and thus immediate actions and intensive studies are needed to control the disease in communities [1]. COVID-19 is the seventh member of the coronavirus (CoV) family, such as MERS-CoV and SARS-CoV [2]. Although SARS-CoV was more deadly, it was much less infectious than COVID-19. There have been no outbreaks of SARS anywhere in the world since 2003. The symptoms of the COVID-19 infection include cough, fever, tiredness, diarrhea, and shortness of breath. Mostly in severe cases, COVID-19 causes pneumonia and death [3]. The primary studies show that the incubation period of COVID-19 is between 3–14 days or longer [4]. Additionally, the average of basic reproduction number ${\mathcal{R}}_{0}$ for COVID-19 is about 2–2.8. The disease may still be infectious in the latent infection period. Studies to date suggest that the virus is very serious and spreads fast from person to person through close contact and respiratory droplets rather than through the air [4]. Table 1 shows the incubation period of several common infectious diseases.

**Table 1:** Incubation period of several common infectious diseases

Disease	Range	Ref.
COVID-19	3–14 days	[4]
Cholera	0.5–4.5 days	[26]
Common cold	1–3 days	[27]
Ebola	1–21 days	[28]
HIV	2–3 weeks to months or longer	[29]
Influenza	1–3 days	–
MERS	2–14 days	[30]
SARS	1–10 days	[31]

Mathematical modeling of the infectious diseases has an important role in the epidemiological aspect of disease control [5–8]. Several epidemic models, with various characteristics, have been described and investigated in the literature. Most of these models are based on susceptible–infected–removed (SIR) model. Casagrandi *et al.* [9] introduced a SIRC model to describe the dynamical behaviors of influenza A by inserting a new compartment, namely cross-immunity (*C*) component^1^ of people who have been recovered after being infected by different strains of the same viral subtype in previous years. The component *C* describes an intermediate state between the susceptible *S* and the recovered *R*. Rihan *et al.* [10] investigated the qualitative behaviors of a fractional-order SIRC model for salmonella bacterial infection. Recently in [11], the authors provided a deterministic SEIR epidemic model of fractional order to describe the dynamics of COVID-19. In other descriptions, quarantine state (*Q*) may be included in the presence of subjects, such as SIRQ models [12].

In fact, stochastic perturbation factors, such as precipitation, absolute humidity, and temperature, have a significant impact on the infection force of all types of virus diseases to humans. Taking this into consideration enables us to present randomness into deterministic biological models to expose the environmental variability effect, whether it is environmental fluctuations in parameters or random noise in the differential systems [13–17]. Moreover, stochastic models give an extra degree of freedom and realism in comparison with their corresponding deterministic models. Stochastic population dynamics perturbed by white noise (or Brownian motion) has been studied extensively by many authors [18–20]. It has been investigated in [21] that an environmental Brownian noise can suppress explosions in population dynamics. Yuan *et al.* [22] discussed the results of stochastic viral infection, immune response dynamics and analyzed the human immuno-deficiency virus infection. In [23], the author investigated the existence results of ergodic distribution for stochastic hepatitis B virus model based on Lyapunov function. In [24], the authors explored the dynamics of SIR epidemic model with environmental fluctuations. Additionally, they calculated a threshold parameter to demonstrate the persistence and extinction of the disease. Recently, Lakshmi *et al.* [25] identified some environmental factors such as geographic location of the countries, the upcoming climate, atmospheric temperature, humidity, sociobiological factors, etc., that influence the global spread of COVID-19.

Up-to-date studies have reported that there are many COVID-19 carriers who are not suffering from the disease. This may be due to cross-immunity of other virus survivors, people who have been recovered from the virus, such as other stains of coronavirus, H1N1, or influenza A. It has been reported in [2] that “*T-cells that target SARS-CoV2, the virus that causes COVID-19, in the blood of people who had recovered from a coronavirus infection.*” Accordingly, in the present paper, we investigate an SIRC epidemic model of cross-immune class for the dynamics of COVID-19 infection among groups. We include time-delay in the transmission terms to represent the incubation period of the virus (the time between infection and symptom onset). We also incorporate white noise type of perturbations to reveal the effect of environmental fluctuations and variability in parameters. Based on the existing literature, this is the first work dealing with the persistence and extinction of a stochastic epidemic model for the COVID-19 infection. We investigate the impact of small and large values of white noise in the persistence and extinction of the disease. We also derive the existence results of stationary distribution and extinction of the disease using a novel combination of stochastic Lyapunov functional. This paper is presented as follows: We provide a stochastic SIRC model with time-delay in Sect. 2. In Sect. 3, we study the existence and uniqueness of a global positive solution for the stochastic delayed SIRC model. In Sect. 4, a stationary distribution and extinction analysis of the underlying model are investigated. Some virtual numerical examples are presented in Sect. 5. Finally, concluding remarks are given in Sect. 6.

## Stochastic SIRC epidemic model

For the spread of the COVID-19 disease in humans, we classify the population into four categories: $S(t)$, $I(t)$, $R(t)$, and $C(t)$ are the proportion of susceptible, infected, recovered, and cross-immune ones at time *t*, respectively. Let $N(t)=S(t)+I(t)+R(t)+C(t)$ be the total population. At this stage, we believe that a SIRC model efficiently describes the mechanism for the spreading of the COVID-19 virus. The classical SIRC model [9, 32] takes the form

1 $$\begin{aligned} \begin{gathered} \dot{S}(t)= \eta \bigl(1-S(t) \bigr)-\xi S(t)I{(t-\tau })+\beta C(t), \\ \dot{I}(t)=\xi S(t)I(t-\tau )+ \sigma \xi C(t)I(t)-(\eta +\alpha )I(t), \\ \dot{R}(t)= (1- \sigma )\xi C(t)I(t)+\alpha I(t)-(\eta +\gamma )R(t), \\ \dot{C}(t)= \gamma R(t)-\xi C(t)I(t)-(\eta +\beta )C(t). \end{gathered} \end{aligned}$$

**Figure 1:**
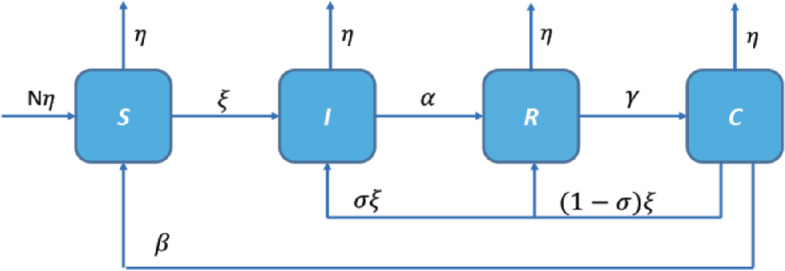
Scheme of SIRC model (1)

We incorporate a discrete time-delay *τ* into the SIRC model to represent the incubation period which is about 3–14 days [4]. All the parameters appearing in the model are nonnegative, see Table 2. In the absence of cross-immunity i.e. $(1-\sigma =0)$, the SIRC model curtails to the SIRS model since the two individuals *S* and *C* become immunologically indistinguishable. Figure 1 shows the scheme of SIRC model.

**Table 2:** Description of the model parameters

Parameters	Description
*η*	Mortality rate in every compartment assumed to be equal to the rate of newborn in the population [9]
*β*	Rate at which the cross-immune population becomes susceptible again
*ξ*	Contact/transmission rate
*σ*	The average reinfection probability of a cross-immune individual
*α*	Recovery rate of the infected population
*γ*	Rate at which the recovered population becomes the cross-immune population and moves from total to partial immunity

Time-delay $\tau >0$ is incorporated in the transmission terms to represent the incubation period of the viral infection, the time between infection and symptom onset. The current studies show that the average/median of incubation period of early confirmed cases of COVID-19 is about 5.5 days, which is similar to SARS-CoV. Presence of time-delay in the model may cause periodic solutions many times for different time-delay values *τ* [33].

Model (1) has a disease-free equilibrium ${\mathcal{E}}_{0}=[1,0,0,0]$ and an endemic equilibrium ${\mathcal{E}}_{+}=[S^{*},I^{*},R^{*},C^{*}]$, where

$$\begin{aligned}& S^{*}= \frac{\eta +\alpha }{\xi }- \frac{\beta \gamma \alpha I^{*}}{[(\eta +\gamma )-(1-\sigma )\gamma ]\xi I^{*}+(\eta +\beta )(\eta +\gamma )}, \\& R^{*}= \frac{\alpha I^{*}(\xi I^{*}+\eta +\beta )}{[(\eta +\gamma )-(1-\sigma )\gamma ]\xi I^{*}+(\eta +\beta )(\eta +\gamma )}, \\& C^{*}= \frac{\gamma \alpha I^{*}}{[(\eta +\gamma )-(1-\sigma )\gamma ]\xi I^{*}+(\eta +\beta )(\eta +\gamma )}, \end{aligned}$$

and $I^{*}$ is a root of quadratic equation $p I^{2}+q I+r=0$, where

$$\begin{aligned} \begin{gathered} p= \eta \xi (\eta +\alpha +\sigma \gamma ), \\ q= \eta \xi \bigl[ \alpha (2\eta +\gamma +\beta )+(\eta +\beta ) ( \eta +\gamma ) +(\eta +\sigma \gamma ) (\eta -\xi ) \bigr], \\ r= \eta (\eta +\beta ) (\eta +\gamma ) (\eta +\alpha ) (1-{ \mathcal{R}}_{0}). \end{gathered} \end{aligned}$$

Here ${\mathcal{R}}_{0}=\frac{\xi }{\eta +\alpha }$ is known as the *basic reproduction number* of the deterministic model.

In fact, there is an increasing indication that superior consistency with some phenomena can be contributed if the effects of environmental noises in the system are taken into account [34]. Epidemic model (1) assumes that the observed dynamics are driven exclusively by internal deterministic cases. Ignoring environmental variability in the modeling may affect the dynamics of the model and transmission of the disease. Accordingly, there is a need to extend the deterministic systems described by differential equations into *stochastic differential equations* (SDEs), where related parameters are modeled as suitable stochastic processes, added to the driving system equations.

From the mathematical and biological point of view, there are some assumptions to incorporate stochastic perturbations into the epidemiological model, such as Markov chain process, parameter perturbations, white noise type, etc. Here, we incorporate white noise type perturbation into model (1), which is proportional to the *S*, *I*, *R*, *C* classes, so that

2 $$\begin{aligned} \begin{aligned} &d{S}(t)= \bigl[\eta \bigl(1-S(t)\bigr)-\xi S(t)I({t-\tau })+\beta C(t)\bigr]\,dt+ \nu _{1} S(t) \,dW_{1}(t), \\ &d{I}(t)=\bigl[\xi S(t)I({t-\tau })+ \sigma \xi C(t)I(t)-(\eta +\alpha )I(t) \bigr]\,dt+ \nu _{2} I(t) \,dW_{2}(t) , \\ &d{R}(t)= \bigl[(1- \sigma )\xi C(t)I(t)+\alpha I(t)-(\eta +\gamma )R(t)\bigr] \,dt +\nu _{3} R(t) \,dW_{3}(t) , \\ &d{C}(t)= \bigl[\gamma R(t)-\xi C(t)I(t)-(\eta +\beta )C(t)\bigr]\,dt +\nu _{4} C(t) \,dW_{4}(t), \end{aligned} \end{aligned}$$

where $W_{1}(t)$, $W_{2}(t)$, $W_{3}(t)$, and $W_{4}(t)$ stand for the independent Brownian motions. $\nu _{1}^{2}$, $\nu _{2}^{2}$, $\nu _{3}^{2}$, and $\nu _{4}^{2}$ represent the intensity of the environmental white noises, $\nu _{i}>0$ ($i=1,2,3,4$) subject to the following initial conditions:

3 $$ \begin{aligned} &S(\theta )=\phi _{1}(\theta ),\quad\quad I(\theta )=\phi _{2}( \theta ), \\ &R(\theta )=\phi _{3}(\theta ),\quad\quad C(\theta )=\phi _{4}(\theta ), \quad \theta \in [-\tau ,0], \\ &\phi _{i}(\theta )\in {\mathcal{C}},\quad i=1,2,3,4, \end{aligned} $$

such that ${\mathcal{C}}$ is a family of Lebesgue integrable functions from $[-\tau ,0]$ into $\mathbb{R}_{+}^{4}$.

## Existence and uniqueness of the positive solution

To investigate the dynamical characteristics of SDDEs (2), the first consideration is to verify if system (2) has a unique global positive solution. As the coefficients of system (2) satisfy the local Lipschitz condition together with the linear growth condition [35, 36], there exists a unique local solution. Now, we need to prove that the solution is positive and global using the Lyapunov analysis method [36].

### Theorem 1

*System* (2) *has a unique positive solution* $(S(t),I(t),R(t),C(t))$*on* $t\geq -\tau $, *and the solution will remain in* ${\mathbb{R}}_{+}^{4}$*for the given initial condition* (3) *with probability one*.

### Proof 1

For any initial value (3), as the coefficients of system (2) satisfy the local Lipschitz condition, so system (2) has a unique local solution $(S(t), I(t), R(t), C(t))$ on $t \in [-\tau , \tau _{e})$ a.s., where $\tau _{e}$ represents the explosion time [36].

Our aim is to show that this solution is global i.e. $\tau _{e} = \infty $ a.s. Assume $n_{0} \geq 1$ to be sufficiently large such that $S(\theta )$, $I(\theta )$, $R(\theta )$, and $C(\theta )$ ($\theta \in [-\tau ,0]$) are lying in the interval $[\frac{1}{n_{0}}, n_{0} ]$. For each $n \geq n_{0}$, $n \in \mathbb{N}$, define the stopping time

$$ \tau _{n}= \inf \biggl\{ t \in [-\tau , \tau _{e}): \min \bigl\{ S(t),I(t),R(t),C(t) \bigr\} \leq \frac{1}{n} \text{ or }\max \bigl\{ S(t),I(t),R(t),C(t)\bigr\} \geq n \biggr\} , $$

we fix $\inf \phi = \infty $ (*ϕ* is the empty set). Apparently, $\tau _{n}$ is increasing as $n \rightarrow \infty $. Assume $\tau _{\infty }= \lim_{n \rightarrow \infty } \tau _{n}$, then $\tau _{\infty } \leq \tau _{e}$ a.s. Therefore, we need to show that $\tau _{\infty }=\infty $ a.s., then $\tau _{e}=\infty $ a.s. and $(S(t),I(t),R(t),C(t))\in \mathbb{R}_{+}^{4}$ a.s. for all $t\geq -\tau $. If it is erroneous, there is a pair $\epsilon \in (0, 1)$ and $\widetilde{T}>0$ such that $P\{\tau _{\infty } \leq \widetilde{T} \}> \epsilon $. Then there is an integer $n_{1} \geq n_{0}$ such that

4 $$ P\{\tau _{n} \leq \widetilde{T} \} \geq \epsilon ,\quad \forall n \geq n_{1}.$$

We define a ${\mathcal{C}}^{2}$-function $\mathcal{V}: \mathbb{R}^{4}_{+} \rightarrow \mathbb{R}_{+}$ as follows:

$$ \begin{aligned} \mathcal{V}(S,I,R,C)&=\biggl(S-\kappa -\kappa \frac{ \ln S}{\kappa }\biggr)+ (I-1- \ln I) \\ &\quad{}+ (R-1-\ln R)+(C-1-\ln C)+ \int _{t}^{t+\tau } \kappa \xi I(s-\tau )\,ds, \end{aligned} $$

where $\kappa >0$ is a constant to be determined. By Ito’s formula, we can obtain

$$\begin{aligned} \begin{aligned} d\mathcal{V}= {}&\mathcal{L}\mathcal{V} \,dt+\nu _{1}(S-\kappa ) \,dW_{1} (t) +\nu _{2}(I-1) \,dW_{2} (t)+\nu _{3}(R-1) \,dW_{3} (t)\\&{}+\nu _{4}(C-1) \,dW_{4} (t), \end{aligned} \end{aligned}$$

where

$$\begin{aligned} \mathcal{L} \mathcal{V}&= \biggl(1-\frac{\kappa }{S}\biggr) \bigl(\eta -\eta S- \xi S I(t- \tau ) + \beta C\bigr)+\biggl(1-\frac{1}{I}\biggr) \bigl(\xi SI(t-\tau )+\sigma \xi CI-( \eta +\alpha )I\bigr) \\ & \quad {} + \biggl(1-\frac{1}{R}\biggr) (\xi CI - \sigma \xi CI+ \alpha I-\eta R - \gamma R)+\biggl(1-\frac{1}{C}\biggr) \bigl(\gamma R-\xi CI \\ & \quad {} -(\eta +\beta )C\bigr)+ \frac{ \kappa \nu _{1}^{2}+\nu _{2}^{2}+\nu _{3}^{2}+\nu _{4}^{2} }{2}+ \kappa \xi I(t)-\kappa \xi I(t-\tau ) \\ &\leq 4\eta +\kappa \eta +\alpha +\beta +\gamma -\eta C-\eta R+\bigl(\xi (1+ \kappa )-\alpha \bigr)I-\eta I-\eta S+ \frac{\kappa \nu _{1}^{2}+\nu _{2}^{2}+\nu _{3}^{2}+\nu _{4}^{2} }{ 2}. \end{aligned}$$

Let $\kappa =\frac{\alpha -\xi }{\xi }$, then we have

5 $$ \begin{aligned} \mathcal{L}\mathcal{V}&\leq 4\eta +\kappa \eta +\alpha + \beta +\gamma + \frac{\kappa \nu _{1}^{2}+\nu _{2}^{2}+\nu _{3}^{2}+\nu _{4}^{2} }{2} \leq \mathcal{M}, \end{aligned} $$

where $\mathcal{M}>0$ is a constant which is independent of $S(t)$, $I(t)$, $R(t)$, and $C(t)$. Therefore,

6 $$\begin{aligned} \begin{aligned}d\mathcal{V}(S,I,R,C)&\leq \mathcal{M}\,dt+\nu _{1}(S- \kappa ) \,dW_{1} (t)+\nu _{2}(I-1) \,dW_{2} (t) \\ &\quad{}+ \nu _{3}(R-1) \,dW_{3} (t)+\nu _{4}(C-1) \,dW_{4} (t). \end{aligned} \end{aligned}$$

Integrating (6) from 0 to $\tau _{n}\wedge \widetilde{T}=\min \{\tau _{n},\widetilde{T}\}$ and then taking the expectation *E* on both sides, we have

7 $$ E\mathcal{V}\bigl(S(\tau _{n}\wedge \widetilde{T}),I( \tau _{n}\wedge \widetilde{T}),R(\tau _{n}\wedge \widetilde{T}),C(\tau _{n}\wedge \widetilde{T})\bigr)\leq E\mathcal{V} \bigl(S(0),I(0),R(0),C(0)\bigr)+\mathcal{M} \widetilde{T}. $$

Let $\Omega _{n}=\{\tau _{n}\leq \widetilde{T}\}$, for $n\geq n_{1}$ and in view of (4), we obtain $P(\Omega _{n})\geq \epsilon $ such that, for every $\omega \in \Omega _{n}$, there is at least one of $S(\tau _{n},\omega )$, $I(\tau _{n},\omega )$, $R(\tau _{n},\omega )$, or $C(\tau _{n},\omega )$ equaling either *n* or $\frac{1}{n}$, and then we obtain

8 $$ \mathcal{V}\bigl(S(\tau _{n}\wedge \widetilde{T}),I(\tau _{n}\wedge \widetilde{T}),R(\tau _{n}\wedge \widetilde{T}),C(\tau _{n}\wedge \widetilde{T})\bigr)\geq (n-1-\ln n) \wedge \biggl(\frac{1}{n}-1-\ln \frac{1}{n}\biggr). $$

According to (7), we get

9 $$\begin{aligned} \begin{aligned} E\mathcal{V}\bigl(S(0),I(0),R(0),C(0)\bigr)+ \mathcal{M} \widetilde{T}&\geq E\bigl[1_{\Omega _{n}(\omega )}\mathcal{V}\bigl(S(\tau _{n}, \omega ),I(\tau _{n},\omega ),R(\tau _{n}, \omega )\bigr),C(\tau _{n}, \omega )\bigr] \\ &\geq \epsilon (n-1-\ln n)\wedge \biggl(\frac{1}{n}-1-\ln \frac{1}{n}\biggr), \end{aligned} \end{aligned}$$

where $1_{\Omega _{n}}$ represents the indicator function of $\Omega _{n}$. Letting $n\rightarrow \infty $ yields

10 $$ \infty >E\mathcal{V}\bigl(S(0),I(0),R(0),C(0)\bigr)+\mathcal{M}\widetilde{T}= \infty , $$

which leads to a contradiction. It can be concluded that $\tau _{\infty }=\infty $ a.s., which proves the theorem.

## Existence of ergodic stationary distribution

Herein, we construct a suitable stochastic Lyapunov function to study the existence of a unique ergodic stationary distribution of the positive solutions to system (2). First, let us assume that $X(t)$ is a regular time-homogenous Markov process in $\mathbb{R}^{d}$ illustrated by the SDDE

11 $$ dX(t)=f\bigl(X(t),X(t-\tau ),t\bigr)\,dt+\sum_{r=1}^{d} g_{r}\bigl(X(t),t\bigr)\,dB_{r}(t). $$

The diffusion matrix of the process $X(t)$ is

$$ \Lambda (x)=\bigl(\lambda _{ij}(x)\bigr),\quad \lambda _{ij}(x)=\sum_{r=1}^{d}g_{r}^{i}(x)g_{r}^{j}(x). $$

### Lemma 1

([37])

*The Markov process* $X(t)$*has a unique ergodic stationary distribution* $\pi (\cdot )$*if there exists a bounded domain* ${\mathcal{U}}\subset \mathbb{R}^{d}$*with regular boundary* Γ, *and*

(i): *there is a positive number* ${\mathcal{K}}$*so that* $\sum_{i,j=1}^{d} \lambda _{ij}(x)\xi _{i}\xi _{j}\geq {\mathcal{K}} \vert \xi \vert ^{2}$, $x\in {\mathcal{U}}$, $\xi \in \mathbb{R}^{d}$.
(ii): *there exists a nonnegative* ${\mathcal{C}}^{2}$-*function* *Ṽ* *so that* ${\mathcal{L}}\widetilde{V}$*is negative for any* $\mathbb{R}^{d} \setminus {\mathcal{U}}$.

Define the reproduction number of the stochastic model as follows:

12 $$ \mathcal{R}_{0}^{s}= \frac{\eta \gamma \xi ^{2}(1-\sigma )}{\hat{\eta }\hat{\alpha }\hat{\gamma } \hat{\beta }}, $$

where $\hat{\eta }=\eta +\frac{\nu _{1}^{2}}{2}$, $\hat{\alpha }=\eta +\alpha +\frac{\nu _{2}^{2}}{2}$, $\hat{\gamma }=\eta +\gamma +\frac{\nu _{3}^{2}}{2}$, and $\hat{\beta }=\eta +\beta +\frac{\nu _{4}^{2}}{2}$.

### Theorem 2

*Assume that* $\mathcal{R}_{0}^{s}>1$*and* $\eta - \frac{\nu _{1}^{2} \vee \nu _{2}^{2} \vee \nu _{3}^{2} \vee \nu _{4}^{2}}{2}>0$, *then for value* $(S(0), I(0), R(0), C(0))\in \mathbb{R}^{4}_{+}$, *system* (2) *has a unique ergodic stationary distribution* $\pi (\cdot )$.

### Proof 2

First, we need to validate conditions $(i)$ and $(\mathit{ii})$ of Lemma 1. To prove condition $(i)$, the diffusion matrix of model (2) is described as follows:

$$ \Lambda = \begin{pmatrix} \nu _{1}^{2} S^{2} & 0 & 0 & 0 \\ 0 & \nu _{2}^{2} I^{2} & 0 & 0 \\ 0 & 0 & \nu _{3}^{2} R^{2} & 0 \\ 0 & 0 & 0 & \nu _{4}^{2} C^{2} \end{pmatrix}. $$

Then the matrix Λ is positive definite for any compact subset of $\mathbb{R}^{4}_{+}$, then condition $(i)$ of Lemma 1 is satisfied.

Next, we prove condition $(\mathit{ii})$. Define ${\mathcal{C}}^{2}$-function $\mathcal{V}: \mathbb{R}^{4}_{+} \rightarrow \mathbb{R}$ as follows:

$$\begin{aligned} \mathcal{V}(S, I, R, C)&=Q \biggl(- \ln S - c_{1} \ln I-c_{2} \ln R-c_{3} \ln C+\xi \int _{t}^{t+\tau }I(s-\tau )\,ds \biggr) \\ &\quad {} -\ln S+\xi \int _{t}^{t+\tau }I(s-\tau )\,ds-\ln R-\ln C+ \frac{1}{\rho +1}(S+I+R+C)^{\rho +1} \\ &=Q \mathcal{V}_{1}+\mathcal{V}_{2}+\mathcal{V}_{3}+ \mathcal{V}_{4}+ \mathcal{V}_{5}, \end{aligned}$$

where $c_{1}= \frac{\eta \gamma \xi ^{2}(1-\sigma )}{\hat{\alpha }^{2}\hat{\gamma }\hat{\beta }}$, $c_{2}= \frac{\eta \gamma \xi ^{2}(1-\sigma )}{\hat{\alpha }\hat{\gamma }^{2}\hat{\beta }}$, and $c_{3}= \frac{\eta \gamma \xi ^{2}(1-\sigma )}{\hat{\alpha }\hat{\gamma }\hat{\beta }^{2}}$. Note that $\mathcal{V}(S, I, R, C)$ is not only continuous, but also tends to +∞ as $(S, I, R, C)$ approaches the boundary of $\mathbb{R}_{+}^{4}$ and $\Vert (S, I, R, C) \Vert \rightarrow \infty $. Therefore, $\mathcal{V}$ must have a minimum point $(S(0), I(0), R(0), C(0))$ in the interior of $\mathbb{R}_{+}^{4}$. We define a ${\mathcal{C}}^{2}$-function $\widetilde{V}:\mathbb{R}_{+}^{4} \rightarrow \mathbb{R}_{+}$ as follows:

13 $$ \begin{aligned} \widetilde{V}(S, I, R, C)&=Q \biggl(- \ln S - c_{1} \ln I-c_{2} \ln R-c_{3} \ln C+\xi \int _{t}^{t+\tau }I(s-\tau )\,ds \biggr) \\ &\quad {} -\ln S+\xi \int _{t}^{t+\tau }I(s-\tau )\,ds-\ln R-\ln C+ \frac{1}{\rho +1}(S+I+R+C)^{\rho +1} \\ &\quad {} -\mathcal{V}\bigl(S(0), I(0), R(0), C(0)\bigr) \\ &:=Q \mathcal{V}_{1}+\mathcal{V}_{2}+\mathcal{V}_{3}+ \mathcal{V}_{4}+ \mathcal{V}_{5}-\mathcal{V}\bigl(S(0), I(0), R(0), C(0)\bigr), \end{aligned} $$

where $(S,I,R,C)\in (\frac{1}{n},n)\times (\frac{1}{n},n)\times ( \frac{1}{n},n)\times (\frac{1}{n},n)$ and $n>1$ is a sufficiently large integer, $\mathcal{V}_{1}=- \ln S - c_{1} \ln I-c_{2} \ln R-c_{3} \ln C+\xi \int _{t}^{t+\tau }I(s-\tau )\,ds$, $\mathcal{V}_{2}=-\ln S+\xi \int _{t}^{t+\tau }I(s-\tau )\,ds$, $\mathcal{V}_{3}=-\ln R$, $\mathcal{V}_{4}=-\ln C$, and $\mathcal{V}_{5}=\frac{1}{\rho +1}(S+I+R+C)^{\rho +1}$. $\rho >1$ is a constant satisfying

$$ \eta -\frac{\rho }{2}\bigl(\nu _{1}^{2} \vee \nu _{2}^{2} \vee \nu _{3}^{2} \vee \nu _{4}^{2}\bigr)>0, $$

and $Q>0$ is a sufficiently large value satisfying the condition

14 $$ -Q\mu +w\leq -2, $$

where $\mu = \frac{\eta \gamma \xi ^{2}(1-\sigma )}{\hat{\alpha }\hat{\gamma }\hat{\beta }}-( \eta +\frac{\nu _{1}^{2}}{2})>0$,

15 $$ \begin{aligned} w={}&\sup_{(S, I, R, C) \in \mathbb{R}^{4}_{+}} \biggl\{ - \frac{1}{4} \biggl[\eta - \frac{\rho }{2}\bigl(\nu _{1}^{2} \vee \nu _{2}^{2} \vee \nu ^{2}_{3} \vee \nu ^{2}_{4} \bigr) \biggr] I^{\rho +1} \\ &{}+3\eta +\gamma + \beta +2\xi I+A+ \frac{\nu _{1}^{2}}{2} +\frac{\nu _{3}^{2}}{2}+ \frac{\nu _{4}^{2}}{2} \biggr\} ,\end{aligned} $$

and

16 $$\begin{aligned} \begin{aligned}[b] A&=\sup_{(S, I, R, C) \in \mathbb{R}_{+}^{4}} \biggl\{ \eta (S+I+R+C)^{ \rho }- \frac{1}{2} \biggl[\eta -\frac{\rho }{2}\bigl(\nu _{1}^{2} \vee \nu _{2}^{2} \vee \nu ^{2}_{3} \vee \nu ^{2}_{4} \bigr)\biggr] (S+I+R+C)^{\rho +1} \biggr\} \\&< \infty .\end{aligned} \end{aligned}$$

Applying Itô’s formula to $\mathcal{V}_{1}$, we have

17 $$ \begin{aligned} \mathcal{L}\mathcal{V}_{1}&= -\frac{\eta }{S}+\eta +\xi I- \frac{\beta C}{S}-\frac{c_{1}\xi SI(t-\tau )}{I} \\ &\quad {}-c_{1} \sigma \xi C+c_{1}( \eta +\alpha )-\frac{c_{2}(1-\sigma )\xi C I}{R}- \frac{c_{2} \alpha I}{R}+c_{2}(\eta +\gamma ) \\ &\quad {} -\frac{c_{3}\gamma R}{C} +c_{3} \xi I+c_{3}(\eta + \beta )+ \frac{\nu _{1}^{2}}{2}+\frac{c_{1}\nu _{2}^{2}}{2}+ \frac{c_{2}\nu _{3}^{2}}{2}+ \frac{c_{3}\nu _{4}^{2}}{2} \\ &\leq -4 \sqrt[4]{\eta \gamma \xi ^{2}(1-\sigma )c_{1}c_{2}c_{3}}+ \eta +\frac{\nu _{1}^{2}}{2}+c_{1}\biggl(\eta +\alpha + \frac{\nu _{2}^{2}}{2} \biggr)+c_{2}\biggl(\eta +\gamma +\frac{\nu _{3}^{2}}{2} \biggr) \\ &\quad {}+c_{3}\biggl( \eta +\beta +\frac{\nu _{4}^{2}}{2}\biggr)+\xi (1+c_{3}) I \\ &\leq - \frac{\eta \gamma \xi ^{2}(1-\sigma )}{\hat{\alpha }\hat{\gamma }\hat{\beta }}+ \eta +\frac{\nu _{1}^{2}}{2}+\xi (1+c_{3}) I \\ &=-\mu +\xi (1+c_{3})I. \end{aligned} $$

Similarly, we can get

18 $$\begin{aligned}& \mathcal{L}\mathcal{V}_{2} = -\frac{\eta }{S} + \eta +\xi I - \frac{\beta C}{S} +\frac{\nu _{1}^{2}}{2}, \end{aligned}$$

19 $$\begin{aligned}& \mathcal{L}\mathcal{V}_{3}= -\frac{(1- \sigma )\xi CI}{R}- \frac{\alpha I}{R}+\eta +\gamma +\frac{\nu _{3}^{2}}{2}, \end{aligned}$$

20 $$\begin{aligned}& \mathcal{L}\mathcal{V}_{4} = -\frac{\gamma R}{C}+ \xi I + \eta + \beta + \frac{\nu _{4}^{2}}{2}, \end{aligned}$$

21 $$\begin{aligned}& \mathcal{L} \mathcal{V}_{5}=(S+I+R+C)^{\rho } \bigl[\eta - \eta (S+I+R+C)\bigr]+ \frac{\rho }{2}(S+I+R+C)^{\rho -1} \\& \hphantom{\mathcal{L} \mathcal{V}_{5}} \quad {} \times \bigl[\nu _{1}^{2} S^{2}+ \nu _{2}^{2} I^{2} + \nu ^{2}_{3} R^{2} + \nu ^{2}_{4} C^{2}\bigr] \\& \hphantom{\mathcal{L} \mathcal{V}_{5}}\leq (S+I+R+C)^{\rho }\bigl[\eta -\eta (S+I+R+C)\bigr]+ \frac{\rho }{2}(S+I+R+C)^{ \rho +1}\bigl(\nu _{1}^{2} \vee \nu _{2}^{2} \vee \nu ^{2}_{3} \vee \nu ^{2}_{4} \bigr) \\& \hphantom{\mathcal{L} \mathcal{V}_{5}}\leq \eta (S+I+R+C)^{\rho }- (S+I+R+C)^{\rho +1}\biggl[\eta - \frac{\rho }{2}\bigl(\nu _{1}^{2} \vee \nu _{2}^{2} \vee \nu ^{2}_{3} \vee \nu ^{2}_{4} \bigr)\biggr] \\& \hphantom{\mathcal{L} \mathcal{V}_{5}}\leq A - \frac{1}{2} \biggl[\eta -\frac{\rho }{2}\bigl(\nu _{1}^{2} \vee \nu _{2}^{2} \vee \nu ^{2}_{3} \vee \nu ^{2}_{4} \bigr) \biggr](S+I+R+C)^{\rho +1} \\& \hphantom{\mathcal{L} \mathcal{V}_{5}}\leq A - \frac{1}{2} \biggl[\eta -\frac{\rho }{2}\bigl(\nu _{1}^{2} \vee \nu _{2}^{2} \vee \nu ^{2}_{3} \vee \nu ^{2}_{4} \bigr)\biggr] \bigl(S^{\rho +1}+I^{\rho +1}+R^{ \rho +1}+C^{\rho +1}\bigr), \end{aligned}$$

where *A* is defined by (16). From equations (17)–(21), we have

$$\begin{aligned} \mathcal{L}\widetilde{V} &\leq -Q\mu +Q \xi (1+c_{3})I - \frac{1}{2} \biggl[\eta -\frac{\rho }{2} \bigl(\nu _{1}^{2} \vee \nu _{2}^{2} \vee \nu ^{2}_{3} \vee \nu ^{2}_{4} \bigr) \biggr] \bigl(S^{\rho +1}+I^{\rho +1}+R^{ \rho +1}+C^{\rho +1} \bigr) \\ &\quad {}-\frac{\eta }{S} + 3\eta -\frac{\beta C}{S} +\frac{\nu _{1}^{2}}{2} - \frac{\alpha I}{R}+ \gamma +\frac{\nu _{3}^{2}}{2}-\frac{\gamma R}{C}+ 2 \xi I +A +\beta + \frac{\nu _{4}^{2}}{2} \\ &\leq -Q\mu +Q\xi (1+c_{3})I - \frac{1}{4} \biggl[\eta - \frac{\rho }{2}\bigl(\nu _{1}^{2} \vee \nu _{2}^{2} \vee \nu ^{2}_{3} \vee \nu ^{2}_{4} \bigr)\biggr] \bigl(S^{\rho +1}+I^{ \rho +1}+R^{\rho +1}+C^{\rho +1} \bigr) \\ &\quad {} -\frac{\eta }{S} -\frac{1}{4}\biggl[\eta -\frac{\rho }{2}\bigl(\nu _{1}^{2} \vee \nu _{2}^{2} \vee \nu ^{2}_{3} \vee \nu ^{2}_{4} \bigr) \biggr]I^{\rho +1}+ 3\eta - \frac{\beta C}{S} +\frac{\nu _{1}^{2}}{2} - \frac{\alpha I}{R}+\gamma + \frac{\nu _{3}^{2}}{2}-\frac{\gamma R}{C} \\ &\quad{} + 2\xi I +A + \beta + \frac{\nu _{4}^{2}}{2}. \end{aligned}$$

For $\epsilon >0$, define a bounded closed set

$$ \mathcal{D}= \biggl\{ (S, I, R, C) \in \mathbb{R}_{+}^{4}: \epsilon \leq S \leq \frac{1}{\epsilon }, \epsilon \leq I \leq \frac{1}{\epsilon }, \epsilon ^{2} \leq R \leq \frac{1}{\epsilon ^{2}}, \epsilon ^{3} \leq C \leq \frac{1}{\epsilon ^{3}} \biggr\} . $$

In the set $\mathbb{R}_{+}^{4} \setminus \mathcal{D}$, let us choose *ϵ* satisfying the following conditions:

22 $$\begin{aligned}& -\frac{\eta }{\epsilon }+ H \leq -1, \end{aligned}$$

23 $$\begin{aligned}& -Q\mu +Q\xi (1+c_{3})\epsilon +w\leq -1, \end{aligned}$$

24 $$\begin{aligned}& -\frac{\alpha }{\epsilon }+H \leq -1, \end{aligned}$$

25 $$\begin{aligned}& -\frac{\gamma }{\epsilon }+H \leq -1, \end{aligned}$$

26 $$\begin{aligned}& - \frac{1}{4} \biggl[\eta -\frac{\rho }{2}\bigl(\nu _{1}^{2} \vee \nu _{2}^{2} \vee \nu ^{2}_{3} \vee \nu ^{2}_{4} \bigr) \biggr] \frac{1}{\epsilon ^{\rho +1}}+H \leq -1, \end{aligned}$$

27 $$\begin{aligned}& - \frac{1}{4} \biggl[\eta -\frac{\rho }{2}\bigl(\nu _{1}^{2} \vee \nu _{2}^{2} \vee \nu ^{2}_{3} \vee \nu ^{2}_{4} \bigr) \biggr] \frac{1}{\epsilon ^{2(\rho +1)}}+H \leq -1, \end{aligned}$$

28 $$\begin{aligned}& - \frac{1}{4} \biggl[\eta -\frac{\rho }{2}\bigl(\nu _{1}^{2} \vee \nu _{2}^{2} \vee \nu ^{2}_{3} \vee \nu ^{2}_{4} \bigr) \biggr] \frac{1}{\epsilon ^{3(\rho +1)}}+H \leq -1, \end{aligned}$$

where

$$\begin{aligned} H ={}&\sup_{(S, I, R, C) \in \mathbb{R}^{4}_{+}} \biggl\{ Q(c_{3}+1) \xi I - \frac{1}{4} \biggl[\eta -\frac{\rho }{2}\bigl(\nu _{1}^{2} \vee \nu _{2}^{2} \vee \nu ^{2}_{3} \vee \nu ^{2}_{4} \bigr) \biggr] I^{\rho +1} \\ &{}+3\eta + \gamma +\beta +2\xi I+A+\frac{\nu _{1}^{2}}{2} + \frac{\nu _{3}^{2}}{2}+ \frac{\nu _{4}^{2}}{2} \biggr\} . \end{aligned}$$

We need to show that $\mathcal{L}\widetilde{V} \leq -1$ for any $(S, I, R, C) \in \mathbb{R}^{4}_{+} \setminus \mathcal{D}$, and $\mathbb{R}^{4}_{+} \setminus \mathcal{D}=\bigcup_{i=1}^{8} \mathcal{D}_{i}$, where

$$\begin{aligned}& \mathcal{D}_{1} =\bigl\{ (S, I, R, C) \in \mathbb{R}^{4}_{+}; 0< S < \epsilon \bigr\} , \\& \mathcal{D}_{2} =\bigl\{ (S, I, R, C) \in \mathbb{R}^{4}_{+}; 0< I < \epsilon \bigr\} , \\& \mathcal{D}_{3} =\bigl\{ (S, I, R, C) \in \mathbb{R}^{4}_{+}; 0< R < \epsilon ^{2}, I \geq \epsilon \bigr\} , \\& \mathcal{D}_{4} =\bigl\{ (S, I, R, C) \in \mathbb{R}^{4}_{+}; 0< C < \epsilon ^{3}, R \geq \epsilon ^{2} \bigr\} , \\& \mathcal{D}_{5} =\biggl\{ (S, I, R, C) \in \mathbb{R}^{4}_{+}; S> \frac{1}{\epsilon } \biggr\} , \\& \mathcal{D}_{6} =\biggl\{ (S, I, R, C) \in \mathbb{R}^{4}_{+}; I> \frac{1}{\epsilon } \biggr\} , \\& \mathcal{D}_{7} =\biggl\{ (S, I, R, C) \in \mathbb{R}^{4}_{+}; R> \frac{1}{\epsilon ^{2}} \biggr\} , \\& \mathcal{D}_{8} =\biggl\{ (S, I, R, C) \in \mathbb{R}^{4}_{+}; C> \frac{1}{\epsilon ^{3}} \biggr\} . \end{aligned}$$

*Case *1*.* For any $(S, I, R, C) \in \mathcal{D}_{1}$, we obtain

$$\begin{aligned} \mathcal{L}\widetilde{V} &\leq -\frac{\eta }{S}+Q(c_{3}+1) \xi I - \frac{1}{4} \biggl[\eta -\frac{\rho }{2}\bigl(\nu _{1}^{2} \vee \nu _{2}^{2} \vee \nu ^{2}_{3} \vee \nu ^{2}_{4} \bigr) \biggr] I^{\rho +1} +3\eta + \gamma +\beta +2\xi I+A \\ &\quad {} +\frac{\nu _{1}^{2}}{2} +\frac{\nu _{3}^{2}}{2}+ \frac{\nu _{4}^{2}}{2} \\ &\leq -\frac{\eta }{S}+ H \\ &\leq -\frac{\eta }{\epsilon }+ H \leq -1, \end{aligned}$$

which is obtained from (22). Therefore, $\mathcal{L}\widetilde{V}\leq -1$ for any $(S,I,R,C)\in D_{1}$.

*Case *2*.* For any $(S, I, R, C) \in \mathcal{D}_{2}$, we have

$$\begin{aligned} \mathcal{L}\widetilde{V} &\leq -Q\mu +Q\xi (1+c_{3})I - \frac{1}{4} \biggl[\eta -\frac{\rho }{2}\bigl(\nu _{1}^{2} \vee \nu _{2}^{2} \vee \nu ^{2}_{3} \vee \nu ^{2}_{4} \bigr) \biggr] I^{\rho +1} +3\eta +\gamma + \beta +2\xi I \\ &\quad {} +A+\frac{\nu _{1}^{2}}{2} +\frac{\nu _{3}^{2}}{2}+ \frac{\nu _{4}^{2}}{2} \\ &\leq -Q\mu +Q\xi (1+c_{3})I+w \\ &\leq -Q\mu +Q\xi (1+c_{3})\epsilon +w < -1, \end{aligned}$$

which follows from (23) and (14). Thus, $\mathcal{L}\widetilde{V}\leq -1$ for any $(S,I,R,C)\in D_{2}$.

*Case *3*.* For any $(S, I, R, C) \in \mathcal{D}_{3}$, we can get

$$\begin{aligned} \mathcal{L}\widetilde{V} &\leq -\frac{\alpha I}{R}+Q(c_{3}+1) \xi I - \frac{1}{4} \biggl[\eta -\frac{\rho }{2}\bigl(\nu _{1}^{2} \vee \nu _{2}^{2} \vee \nu ^{2}_{3} \vee \nu ^{2}_{4} \bigr) \biggr] I^{\rho +1} +3\eta + \gamma +\beta +2\xi I+A \\ &\quad {} +\frac{\nu _{1}^{2}}{2} +\frac{\nu _{3}^{2}}{2}+ \frac{\nu _{4}^{2}}{2} \\ &\leq -\frac{\alpha \epsilon }{\epsilon ^{2}}+H \leq -1, \end{aligned}$$

which follows from (24). Consequently, $\mathcal{L}\widetilde{V}\leq -1$ for any $(S,I,R,C)\in D_{3}$.

*Case *4*.* For any $(S, I, R, C) \in \mathcal{D}_{4}$, it yields

$$\begin{aligned} \mathcal{L}\widetilde{V} &\leq -\frac{\gamma R}{C}+Q(c_{3}+1) \xi I - \frac{1}{4} \biggl[\eta -\frac{\rho }{2}\bigl(\nu _{1}^{2} \vee \nu _{2}^{2} \vee \nu ^{2}_{3} \vee \nu ^{2}_{4} \bigr) \biggr] I^{\rho +1} +3\eta + \gamma +\beta +2\xi I+A \\ &\quad {} +\frac{\nu _{1}^{2}}{2} +\frac{\nu _{3}^{2}}{2}+ \frac{\nu _{4}^{2}}{2} \\ &\leq -\frac{\gamma \epsilon ^{2}}{\epsilon ^{3}}+H \leq -1, \end{aligned}$$

which is obtained from (25). Thus, $\mathcal{L} \widetilde{V}\leq -1$ for any $(S,I,R,C)\in D_{4}$.

*Case *5*.* If $(S, I, R, C) \in \mathcal{D}_{5}$, we have

$$\begin{aligned} \mathcal{L}\widetilde{V} &\leq - \frac{1}{4} \biggl[\eta - \frac{\rho }{2}\bigl(\nu _{1}^{2} \vee \nu _{2}^{2} \vee \nu ^{2}_{3} \vee \nu ^{2}_{4} \bigr) \biggr] S^{\rho +1} +Q(c_{3}+1) \xi I \\ &\quad {}- \frac{1}{4} \biggl[ \eta -\frac{\rho }{2}\bigl(\nu _{1}^{2} \vee \nu _{2}^{2} \vee \nu ^{2}_{3} \vee \nu ^{2}_{4} \bigr) \biggr] I^{\rho +1} +3\eta \\ &\quad {} +\gamma +\beta +2\xi I+A+\frac{\nu _{1}^{2}}{2} + \frac{\nu _{3}^{2}}{2}+ \frac{\nu _{4}^{2}}{2} \\ &\leq - \frac{1}{4} \biggl[\eta -\frac{\rho }{2}\bigl(\nu _{1}^{2} \vee \nu _{2}^{2} \vee \nu ^{2}_{3} \vee \nu ^{2}_{4} \bigr) \biggr] \frac{1}{\epsilon ^{\rho +1}}+H \leq -1, \end{aligned}$$

which is obtained from (26). Then we can obtain $\mathcal{L}\widetilde{V}\leq -1$ for any $(S,I,R,C)\in D_{5}$.

*Case *6*.* If $(S, I, R, C) \in \mathcal{D}_{6}$, we get

$$\begin{aligned} \mathcal{L}\widetilde{V} &\leq - \frac{1}{4} \biggl[\eta - \frac{\rho }{2}\bigl(\nu _{1}^{2} \vee \nu _{2}^{2} \vee \nu ^{2}_{3} \vee \nu ^{2}_{4} \bigr) \biggr] I^{\rho +1}+Q(c_{3}+1) \xi I \\ &\quad {}- \frac{1}{4} \biggl[ \eta -\frac{\rho }{2}\bigl(\nu _{1}^{2} \vee \nu _{2}^{2} \vee \nu ^{2}_{3} \vee \nu ^{2}_{4} \bigr) \biggr] I^{\rho +1} +3\eta \\ &\quad {} +\gamma +\beta +2\xi I+A+\frac{\nu _{1}^{2}}{2} + \frac{\nu _{3}^{2}}{2}+ \frac{\nu _{4}^{2}}{2} \\ &\leq - \frac{1}{4} \biggl[\eta -\frac{\rho }{2}\bigl(\nu _{1}^{2} \vee \nu _{2}^{2} \vee \nu ^{2}_{3} \vee \nu ^{2}_{4} \bigr) \biggr] \frac{1}{\epsilon ^{\rho +1}}+H \leq -1, \end{aligned}$$

which is obtained from (26). Hence, $\mathcal{L} \widetilde{V}\leq -1$ for any $(S,I,R,C)\in D_{6}$.

*Case *7*.* If $(S, I, R, C) \in \mathcal{D}_{7}$, it yields

$$\begin{aligned} \mathcal{L}\widetilde{V} &\leq - \frac{1}{4} \biggl[\eta - \frac{\rho }{2}\bigl(\nu _{1}^{2} \vee \nu _{2}^{2} \vee \nu ^{2}_{3} \vee \nu ^{2}_{4} \bigr) \biggr] R^{\rho +1}+Q(c_{3}+1) \xi I \\ &\quad {}- \frac{1}{4} \biggl[ \eta -\frac{\rho }{2}\bigl(\nu _{1}^{2} \vee \nu _{2}^{2} \vee \nu ^{2}_{3} \vee \nu ^{2}_{4} \bigr) \biggr] I^{\rho +1} +3\eta \\ &\quad {} +\gamma +\beta +2\xi I+A+\frac{\nu _{1}^{2}}{2} + \frac{\nu _{3}^{2}}{2}+ \frac{\nu _{4}^{2}}{2} \\ &\leq - \frac{1}{4} \biggl[\eta -\frac{\rho }{2}\bigl(\nu _{1}^{2} \vee \nu _{2}^{2} \vee \nu ^{2}_{3} \vee \nu ^{2}_{4} \bigr) \biggr] \frac{1}{\epsilon ^{2\rho +2}}+H \leq -1, \end{aligned}$$

which is obtained from (27). Hence, $\mathcal{L} \widetilde{V}\leq -1$ for any $(S,I,R,C)\in D_{7}$.

*Case *8*.* If $(S, I, R, C) \in \mathcal{D}_{8}$, we can see that

$$\begin{aligned} \mathcal{L}\widetilde{V} &\leq - \frac{1}{4} \biggl[\eta - \frac{\rho }{2}\bigl(\nu _{1}^{2} \vee \nu _{2}^{2} \vee \nu ^{2}_{3} \vee \nu ^{2}_{4} \bigr) \biggr] C^{\rho +1}+Q(c_{3}+1) \xi I \\ &\quad {}- \frac{1}{4} \biggl[ \eta -\frac{\rho }{2}\bigl(\nu _{1}^{2} \vee \nu _{2}^{2} \vee \nu ^{2}_{3} \vee \nu ^{2}_{4} \bigr) \biggr] I^{\rho +1} +3\eta \\ &\quad {} +\gamma +\beta +2\xi I+A+\frac{\nu _{1}^{2}}{2} + \frac{\nu _{3}^{2}}{2}+ \frac{\nu _{4}^{2}}{2} \\ &\leq - \frac{1}{4} \biggl[\eta -\frac{\rho }{2}\bigl(\nu _{1}^{2} \vee \nu _{2}^{2} \vee \nu ^{2}_{3} \vee \nu ^{2}_{4} \bigr) \biggr] \frac{1}{\epsilon ^{3\rho +3}}+H \leq -1, \end{aligned}$$

which is obtained from (28). Therefore, $\mathcal{L}\widetilde{V}\leq -1$ for any $(S,I,R,C)\in D_{8}$.

Clearly, condition $(\mathit{ii})$ of Lemma 1 holds. Therefore, we conclude that system (2) identifies a unique stationary distribution $\pi (\cdot )$.

### Extinction

In order to show the extinction of the disease, we go through the following lemmas.

#### Lemma 2

([38])

*Let* $M=\{M_{t}\}_{t\geq 0}$*be a real*-*valued continuous local martingale vanishing at* $t=0$. *Then*

$$ \lim_{t \rightarrow \infty } \langle M,M \rangle _{t} =\infty \quad \textit{a.s.} \quad \Rightarrow \quad \lim_{t \rightarrow \infty } \frac{M_{t}}{\langle M,M \rangle _{t}}=0\quad \textit{a.s.}, $$

*and also*

$$ \lim \sup_{t\rightarrow \infty } \frac{\langle M,M \rangle _{t}}{t}< \infty \quad \textit{a.s.} \quad \Rightarrow \quad \lim_{t\rightarrow \infty } \frac{M_{t}}{t}=0 \quad \textit{a.s.}, $$

*where* $\langle M,M \rangle _{t}$*denotes the quadratic variation of M*.

#### Lemma 3

(see Lemmas 2.1 and 2.2 in [39])

*Let* $(S(t), I(t), R(t), C(t))$*be the solution of* (2) *with any* $(S(0), I(0), R(0), C(0)) \in \mathbb{R}_{+}^{4}$, *then*

$$ \lim_{t \rightarrow \infty } \frac{S(t)}{t}=0, \quad\quad \lim _{t \rightarrow \infty } \frac{I(t)}{t}=0, \quad\quad \lim _{t \rightarrow \infty } \frac{R(t)}{t}=0, \quad\quad \lim _{t \rightarrow \infty } \frac{C(t)}{t}=0, \quad \textit{a.s.} $$

*Furthermore*, *if* $\eta > \frac{\nu _{1}^{2} \vee \nu _{2}^{2} \vee \nu _{3}^{2} \vee \nu _{4}^{2}}{2}$, *then*

$$\begin{aligned}& \lim_{t \rightarrow \infty } \frac{\int _{0}^{t}S(s)\,dW_{1}(s)}{t}=0, \quad \quad \lim _{t \rightarrow \infty } \frac{\int _{0}^{t}I(s)\,dW_{2}(s)}{t}=0, \quad \quad \lim _{t \rightarrow \infty } \frac{\int _{0}^{t}R(s)\,dW_{3}(s)}{t}=0, \\& \lim_{t \rightarrow \infty } \frac{\int _{0}^{t}C(s)\,dW_{4}(s)}{t}=0, \quad \textit{a.s.} \end{aligned}$$

#### Theorem 3

*If* $\mathcal{R}_{0}^{s}<1$*and* $\eta > \frac{\nu _{1}^{2} \vee \nu _{2}^{2} \vee \nu _{3}^{2} \vee \nu _{4}^{2}}{2}$, *then the solution of* (2) *satisfies the following*: $\lim_{t \rightarrow \infty } \sup \frac{1}{t}\ln ( \alpha (I(t)+C(t))+(\eta +\alpha ) R(t)) \leq \xi - \frac{1}{2(\alpha )^{2}} \{ \alpha ^{2} \frac{\nu ^{2}_{2}}{2} \wedge (\eta (\eta +\alpha +\gamma )+ (\eta +\alpha )^{2} \frac{\nu _{3}^{2}}{2}) \wedge \alpha ^{2}(\eta +\beta + \frac{\nu _{4}^{2}}{2}) \} <0$*and* $\lim_{t \rightarrow \infty } \langle S \rangle =1$*a*.*s*.

#### Proof 3

Define $U(t)=\alpha (I(t)+C(t))+(\eta +\alpha ) R(t)$, taking Ito’s formula, we can get

29 $$\begin{aligned}& d \ln U(t) \\& \quad = \biggl\{ \frac{1}{\alpha (I+C)+(\eta +\alpha ) R} \bigl[ \alpha \xi S{I(t-\tau )}- \alpha (\eta + \beta )C-\bigl(\eta ^{2}+\eta \alpha + \eta \gamma \bigr)R \bigr] \\& \quad \quad {} - \frac{ [\alpha ^{2} \nu ^{2}_{2}I^{2}+(\eta +\alpha )^{2} \nu ^{2}_{3} R^{2}+\alpha ^{2} \nu ^{2}_{4} C^{2} ]}{2(\alpha (I+C)+(\eta +\alpha ) R)^{2}} \biggr\} \,dt + \frac{\alpha \nu _{2} I}{\alpha (I+C)+(\eta +\alpha ) R} \,dW_{2}(t) \\& \quad \quad {} + \frac{(\eta +\alpha ) \nu _{3} R}{\alpha (I+C)+(\eta +\alpha ) R}\,dW_{3}(t)+ \frac{\alpha \nu _{4} C}{\alpha (I+C)+(\eta +\alpha ) R} \,dW_{4}(t) \\& \quad \leq \xi S \,dt- \frac{1}{(\alpha (I+C)+(\eta +\alpha ) R)^{2}} \biggl\{ \alpha ^{2} \frac{\nu ^{2}_{2}}{2}I^{2}+ \alpha ^{2}\biggl(\eta + \beta + \frac{\nu _{4}^{2}}{2}\biggr) C^{2} +\biggl(\eta (\eta +\alpha +\gamma ) \\& \quad \quad {} +(\eta +\alpha )^{2} \frac{\nu _{3}^{2}}{2} \biggr)R^{2} \biggr\} \,dt + \frac{\alpha \nu _{2} I}{\alpha (I+C)+(\eta +\alpha ) R}\,dW_{2}(t) \\& \quad \quad {} + \frac{(\eta +\alpha ) \nu _{3} R}{\alpha (I+C)+(\eta +\alpha ) R}\,dW_{3}(t)+ \frac{\alpha \nu _{4} C}{\alpha (I+C)+(\eta +\alpha ) R} \,dW_{4}(t) \\& \quad \leq \xi S \,dt- \frac{1}{2(\alpha )^{2}} \biggl\{ \alpha ^{2} \frac{\nu ^{2}_{2}}{2}\wedge \biggl(\eta (\eta +\alpha +\gamma )+ (\eta + \alpha )^{2} \frac{\nu _{3}^{2}}{2}\biggr) \wedge \alpha ^{2}\biggl(\eta +\beta + \frac{\nu _{4}^{2}}{2}\biggr) \biggr\} \,dt \\& \quad \quad {} + \frac{\alpha \nu _{2} I}{\alpha (I+C)+(\eta +\alpha ) R}\,dW_{2}(t)+ \frac{(\eta +\alpha ) \nu _{3} R}{\alpha (I+C)+(\eta +\alpha ) R} \,dW_{3}(t) \\& \quad \quad {} + \frac{\alpha \nu _{4} C}{\alpha (I+C)+(\eta +\alpha ) R}\,dW_{4}(t). \end{aligned}$$

From model (2), we have

30 $$\begin{aligned} d\bigl(S(t)+I(t)+R(t)+C(t)\bigr)&= \bigl[\eta -\eta \bigl(S(t)+I(t)+R(t)+C(t) \bigr) \bigr]\,dt + \nu _{1} S(t) \,dW_{1}(t) \\ & \quad {} + \nu _{2} I(t) \,dW_{2}(t) + \nu _{3} R(t) \,dW_{3}(t)+\nu _{4} C(t)\,dW_{4}(t). \end{aligned}$$

Taking integration of (30) from 0 to *t*, we obtain

31 $$ \bigl\langle S(t)+I(t)+R(t)+C(t) \bigr\rangle =1+\psi _{1}(t), $$

where

32 $$ \begin{aligned} \psi _{1}(t)&=\frac{1}{\eta } \biggl[ \frac{1}{t}\bigl(S(0)+I(0)+R(0)+C(0)\bigr) \\ &\quad {}- \frac{1}{t} \bigl(S(t)+I(t)+R(t)+C(t)\bigr) + \frac{\nu _{1}\int _{0}^{t}S(s)\,dW_{1}(s)}{t} \\ &\quad {} + \frac{\nu _{2}\int _{0}^{t}I(s)\,dW_{2}(s)}{t} + \frac{\nu _{3}\int _{0}^{t}R(s)\,dW_{3}(s)}{t}+ \frac{\nu _{4}\int _{0}^{t}C(s)\,dW_{4}(s)}{t} \biggr]. \end{aligned} $$

By Lemmas 2 and 3, we can easily obtain that

$$ \lim_{t\rightarrow \infty } \psi _{1}(t)=0\quad \text{a.s.} $$

Therefore, by taking the superior limit on both sides of (31), we obtain

33 $$ \lim_{t \rightarrow \infty } \sup \bigl\langle S(t)+I(t)+R(t)+C(t)\bigr\rangle =1 \quad \text{a.s.} $$

Integrating (29) from 0 to *t*, we obtain

34 $$ \begin{aligned} \frac{\ln U(t)}{t}\leq {}&\xi -\frac{1}{2(\alpha )^{2}} \biggl\{ \alpha ^{2} \frac{\nu ^{2}_{2}}{2}\wedge \biggl(\eta (\eta +\alpha +\gamma )+ ( \eta + \alpha )^{2} \frac{\nu _{3}^{2}}{2}\biggr) \wedge \alpha ^{2}\biggl(\eta +\beta + \frac{\nu _{4}^{2}}{2}\biggr) \biggr\} \\ &{}+\psi _{2}(t), \end{aligned} $$

where

$$\begin{aligned} \psi _{2}(t)&=\frac{\ln U(0)}{t}+ \frac{\alpha \nu _{2}}{t} \int _{0}^{t} \biggl( \frac{ I(s)}{\alpha (I(s)+C(s))+(\eta +\alpha ) R(s)} \,dW_{2}(s) \biggr) \\ &\quad {} +\frac{(\eta +\alpha ) \nu _{3}}{t} \int _{0}^{t} \biggl( \frac{ R(s)}{\alpha (I(s)+C(s))+(\eta +\alpha ) R(s)} \,dW_{3}(s) \biggr) \\ & \quad {} +\frac{\alpha \nu _{4}}{t} \int _{0}^{t} \biggl( \frac{ C(s)}{\alpha (I(s)+C(s))+(\eta +\alpha ) R(s)} \,dW_{4}(s) \biggr). \end{aligned}$$

In the same manner, by Lemmas 2 and 3, we have

$$ \lim_{t\rightarrow \infty } \psi _{2}(t)=0\quad \text{a.s.} $$

Since $\mathcal{R}_{0}^{s}<1$, therefore, by taking the superior limit of both sides of (34), we have

35 $$ \begin{aligned} & \lim_{t \rightarrow \infty } \sup \frac{\ln U(t)}{t} \\ &\quad \leq \xi - \frac{1}{2(\alpha )^{2}} \biggl\{ \alpha ^{2} \frac{\nu ^{2}_{2}}{2} \wedge \biggl(\eta (\eta +\alpha +\gamma )+ (\eta +\alpha )^{2} \frac{\nu _{3}^{2}}{2}\biggr) \wedge \alpha ^{2}\biggl(\eta +\beta + \frac{\nu _{4}^{2}}{2}\biggr) \biggr\} \\ &\quad < 0, \end{aligned} $$

which implies that $\lim_{t \rightarrow \infty }I(t)=0$, $\lim_{t \rightarrow \infty }R(t)=0$, $\lim_{t \rightarrow \infty }C(t)=0$ a.s., which confirms that the disease *I* can die out with probability one.

It is easy, by using (33) and (35), to show that $\lim_{t \rightarrow \infty } \langle S\rangle =1$ a.s.

## Numerical simulations and discussions

Numerical simulations are given to validate our theoretical results through Euler–Maruyama method for SDDEs reported in [40, 41] to numerically solve SDDEs (2).

The discretization transformation takes the form

36 $$\begin{aligned} \begin{aligned} &S_{j+1}= S_{j}+ \bigl[\eta (1-S_{j})-\xi S_{j}I_{j-m}+\beta C_{j} \bigr] \Delta t+\nu _{1} S_{j}\sqrt{\Delta t}\zeta _{1, j}, \\ &I_{j+1}=I_{j}+\bigl[\xi S_{j}I_{j-m}+ \sigma \xi C_{j}I_{j}-(\eta + \alpha )I_{j}\bigr] \Delta t+ \nu _{2} I_{j} \sqrt{\Delta t}\zeta _{2, j}, \\ &R_{j+1}= R_{j}+\bigl[(1- \sigma )\xi C_{j}I_{j}+ \alpha I_{j}-(\eta + \gamma )R_{j}\bigr]\Delta t +\nu _{3} R_{j}\sqrt{\Delta t}\zeta _{3, j}, \\ &C_{j+1}= C_{j}+\bigl[\gamma R_{j}-\xi C_{j}I_{j}-(\eta +\beta )C_{j}\bigr] \Delta t +\nu _{4} C_{j}\sqrt{\Delta t}\zeta _{4, j}. \end{aligned} \end{aligned}$$

The independent Gaussian random variables denoted as $\zeta _{i, j}$ ($i=1, 2, 3, 4$), which follow the distribution $N(0, 1)$, the time-delay is defined as $\tau =m\Delta t$, *m* is an integer, and the step size Δ*t*. Let $\nu _{i}>0$, ($i=1, 2, 3, 4$) be the white noise values.

### Example 1

**Figure 2:**
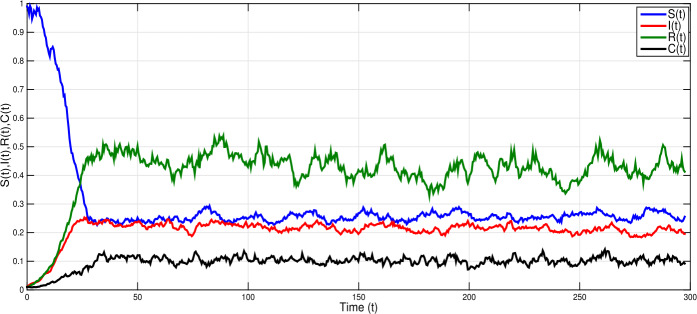
Shows numerical simulations of stochastic model (2) when ${\mathcal{R}}_{0}^{s}=1.3>1$, with $\eta =0.09$, $\xi =1.3$, $\beta =0.05$, $\sigma =0.9$, $\alpha =0.36$, $\gamma =0.1$; $\tau =1$ and white noises $\nu _{1}=0.1$, $\nu _{2}=0.09$, $\nu _{3}=0.09$, $\nu _{4}=0.07$. The model has a unique ergodic stationary distribution and the infection is persistent

Consider model (2) with white noise values $\nu _{1}=0.1$, $\nu _{2}=0.09$, $\nu _{3}=0.09$, $\nu _{4}=0.07$ and parameter values $\eta =0.09$, $\xi =1.3$, $\beta =0.05$, $\sigma =0.9$, $\gamma =0.1$, $\alpha =0.36$, $\tau =1.2$. Simple calculation leads to $\mathcal{R}^{s}_{0}= \frac{\eta \gamma \xi ^{2}(1-\sigma )}{\hat{\eta }\hat{\alpha }\hat{\gamma }\hat{\beta }}=1.3>1$ and $\eta - \frac{\nu _{1}^{2} \vee \nu _{2}^{2} \vee \nu _{3}^{2} \vee \nu _{4}^{2}}{2}=0.087>0$. Therefore, the conditions of Theorem 2 hold. Based on Theorem 2, there is a unique ergodic stationary distribution $\pi (\cdot )$ of model (2). Thus, the disease *I* is persistent; see Fig. 2.

### Example 2

**Figure 3:**
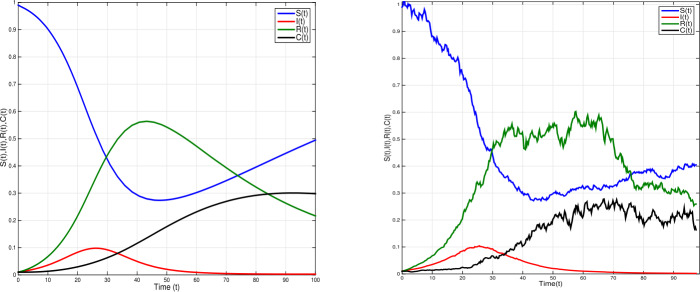
Time domain behaviors of solutions of SDDEs model (2) (right) and the corresponding deterministic model (1) (left) when ${\mathcal{R}}_{0}^{s}=0.38<1$, with $\eta =0.0005$, $\xi =0.6$, $\beta =0.01$, $\sigma =0.12$, $\alpha =0.3$, $\gamma =0.02$; $\tau =1.4$ and white noises $\nu _{1}=\nu _{2}=0.02$, $\nu _{3}=0.01$, $\nu _{4}=0.02$. The infection dies out with probability one

**Figure 4:**
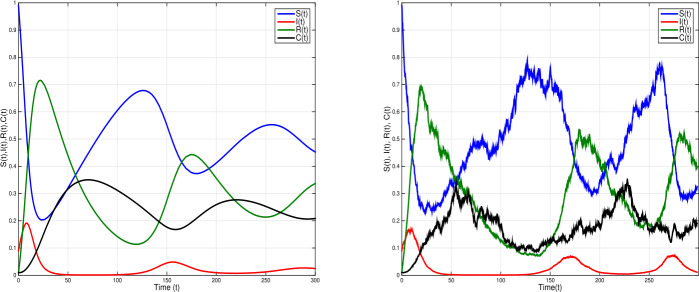
Time domain behaviors of SDDEs model (2) (right) and corresponding deterministic model (1) (left) when ${\mathcal{R}}_{0}^{s}=0.38<1$, with $\eta =0.0005$, $\xi =0.6$, $\beta =0.01$, $\sigma =0.12$, $\alpha =0.3$, $\gamma =0.02$; $\tau =2.5$ and white noises $\nu _{1}=0.02$, $\nu _{2}=0.2$, $\nu _{3}=0.02$, $\nu _{4}=0.2$. The figure shows a periodic outbreak due to the time-delay *τ*

Given model (2) with parameter values $\eta =0.0005$; $\xi =0.6$; $\beta =0.01$; $\sigma =0.12$; $\alpha =0.3$; $\gamma =0.02$, $\tau =1.4$ and white noises $\nu _{1}=0.02$, $\nu _{2}=0.02$, $\nu _{3}=0.01$, $\nu _{4}=0.2$. We obtain $\mathcal{R}^{s}_{0}= \frac{\eta \gamma \xi ^{2}(1-\sigma )}{\hat{\eta }\hat{\alpha }\hat{\gamma }\hat{\beta }}=0.38<1$ and $\eta - \frac{\nu _{1}^{2} \vee \nu _{2}^{2} \vee \nu _{3}^{2} \vee \nu _{4}^{2}}{2}=-0.0195<0$. In this case, the conditions of Theorem 2 are not satisfied. From Fig. 3, we can clearly find that the disease goes to extinction. In Fig. 4 time-delay is increased to $\tau =2.5$, with white noises $\nu _{1}=0.01$, $\nu _{2}=0.2$, $\nu _{3}=0.02$, $\nu _{4}=0.03$, other parameter values are the same as in Fig. 3. Therefore $\mathcal{R}_{0}^{s}<1$ and $\eta - \frac{\nu _{1}^{2} \vee \nu _{2}^{2} \vee \nu _{3}^{2} \vee \nu _{4}^{2}}{2}=-0.0445<0$. The conditions of Theorem 2 are not satisfied. Figure 4 shows a periodic outbreak due to the time-delay *τ*. However, the infection dies out with time with bigger white noise.

### Example 3

**Figure 5:**
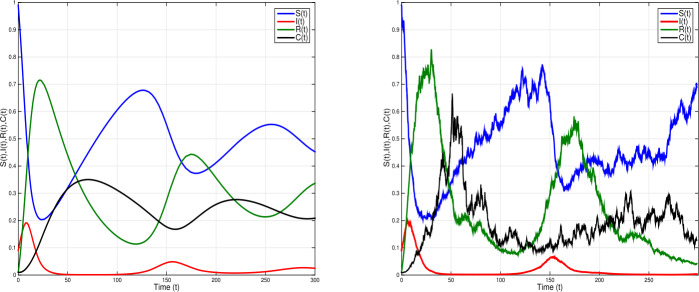
Simulations of stochastic model (2) (right) and the corresponding deterministic model (1) (left) when ${\mathcal{R}}_{0}^{s}=0.38<1$, with $\eta =0.0005$, $\xi =0.6$, $\beta =0.01$, $\sigma =0.12$, $\alpha =0.3$, $\gamma =0.02$; $\tau =2.5$ and white noises $\nu _{1}=0.2$, $\nu _{2}=0.2$, $\nu _{3}=0.1$, $\nu _{4}=0.2$. The deterministic model shows a periodic outbreak due to the time-delay *τ*. The infection dies out with time when white noise is large

To further explain the impact time-delay and white noises on system (2), we choose $\tau =2.5$ and parameter values $\eta =0.0005$; $\xi =0.6$; $\beta =0.01$; $\sigma =0.12$; $\alpha =0.3$; $\gamma =0.02$, and white noises $\nu _{1}=0.2$, $\nu _{2}=0.2$, $\nu _{3}=0.1$, $\nu _{4}=0.3$, such that $\mathcal{R}^{s}_{0}= \frac{\eta \gamma \xi ^{2}(1-\sigma )}{\hat{\eta }\hat{\alpha }\hat{\gamma }\hat{\beta }}=0.38<1$ and $\eta - \frac{\nu _{1}^{2} \vee \nu _{2}^{2} \vee \nu _{3}^{2} \vee \nu _{4}^{2}}{2}=-0.045<0$. Thus, the conditions of Theorem 2 are not satisfied. Figure 5 shows a periodic outbreak due to the time-delay *τ* when the white noise increased the periodicity of the outbreak decreased. The infection dies out with time as white noise increases.

### Example 4

**Figure 6:**
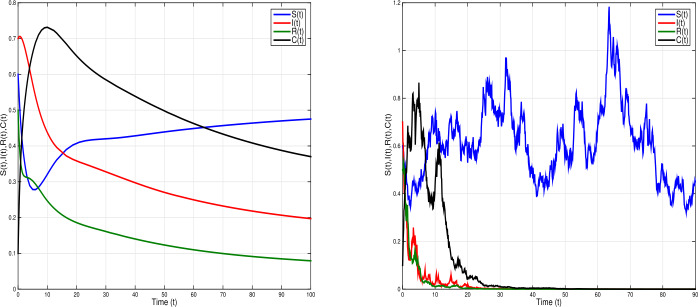
Time domain behaviors of SDDEs model (2) (right) and corresponding deterministic model (1) (left), where $\tau =1$, when ${\mathcal{R}}_{0}=1.78>1$, the infection persists in the deterministic model; when ${\mathcal{R}}_{0}^{s}=0.75<1$, the infection dies out in the stochastic model. With parameter values $\eta =0.02$, $\xi =0.5$, $\beta =0.1$, $\sigma =0.2$, $\alpha =0.26$, $\gamma =1$ and white noises $\nu _{1}=0.13$, $\nu _{2}=0.54$, $\nu _{3}=0.26$, $\nu _{4}=0.75$

In order to show the impact of random perturbation, with $\tau =1$, we increase the white noise values $\nu _{1}=0.13$, $\nu _{2}=0.54$, $\nu _{3}=0.26$, $\nu _{4}=0.75$ with parameter values $\eta =0.02$; $\xi =0.5$; $\beta =0.1$; $\sigma =0.2$; $\alpha =0.26$; $\gamma =1$. Thus, $\mathcal{R}^{s}_{0}= \frac{\eta \gamma \xi ^{2}(1-\sigma )}{\hat{\eta }\hat{\alpha }\hat{\gamma }\hat{\beta }}=0.75<1<1.78= \frac{\xi }{\alpha +\eta }=\mathcal{R}_{0}$, and $\eta - \frac{\nu _{1}^{2} \vee \nu _{2}^{2} \vee \nu _{3}^{2} \vee \nu _{4}^{2}}{2}=0.0115>0$. Therefore, the conditions of Theorem 3 hold, and disease dies out exponentially with probability one. However, the disease persists with deterministic model; see Fig. 6.

### Example 5

**Figure 7:**
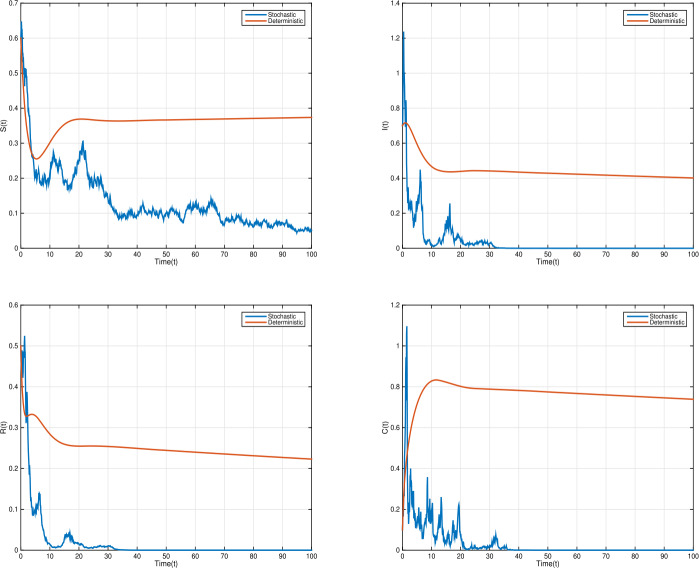
Time response of solutions for model (2) (right) and corresponding deterministic model (1) (left), when ${\mathcal{R}}_{0}=1.78>1$, the infection persists in the deterministic model; when ${\mathcal{R}}_{0}^{s}=0.75<1$, the infection dies out in the stochastic model. With parameter values $\eta =0.02$, $\xi =0.5$, $\beta =0.1$, $\sigma =0.2$, $\alpha =0.26$, $\gamma =1$ and white noises $\nu _{1}=0.13$, $\nu _{2}=0.54$, $\nu _{3}=0.26$, $\nu _{4}=0.75$

Consider the same parameter values of Example 4, but with time-delay $\tau =0$. Thus, according to Theorem 3, the disease dies out exponentially with probability one; see Fig. 7. Therefore, the smaller values of white noise ensure the existence of unique stationary distribution, which gives the persistence of the disease; while larger values of white noise can lead to disease extinction.

### Remark 1

Given the deterministic SIRC model (1), if the basic reproduction number $\mathcal{R}_{0} =\frac{\xi }{\alpha +\eta }<1 $, then the disease-free equilibrium point is globally asymptotically stable; whereas if $\mathcal{R}_{0} > 1$, the unique endemic equilibrium point is globally asymptotically stable. Repeated outbreaks of the infection can occur due to the time-delay in the transmission terms. In our stochastic SIRC model (2), if $\mathcal{R}_{0}^{s}= \frac{\eta \gamma \xi ^{2}(1-\sigma )}{\hat{\eta }\hat{\alpha }\hat{\gamma }\hat{\beta }}<1< \mathcal{R}_{0}$ and $\eta > \frac{\nu _{1}^{2} \vee \nu _{2}^{2} \vee \nu _{3}^{2} \vee \nu _{4}^{2}}{2}$, the stochastic model (2) has disease extinction with probability one, and for $\mathcal{R}_{0}^{s}>1$, the model has a unique ergodic stationary distribution. See Figs. 5, 6, and 7.

## Conclusion

In this work, we provided a stochastic SIRC epidemic model with time-delay for the new strain coronavirus COVID-19. The stochastic components, due to environmental variability, are incorporated in the model as Gaussian white noise. We established some sufficient conditions for persistence and extinction in the mean of the disease. The model has a unique stationary distribution which is ergodic if the intensity of white noise is small. Introduction of noise in the deterministic SIRC model modifies the basic reproductive number ${\mathcal{R}}_{0}$ giving rise to a new threshold quantity ${\mathcal{R}}_{0}^{s}$. It has been proved that the disease dies out if ${\mathcal{R}}_{0}^{s} < 1<{\mathcal{R}}_{0}$. On the other hand, if ${\mathcal{R}}_{0}^{s}>1$ and ${\mathcal{R}}_{0}>1$, the disease persists with both models, but with different behaviors. In other words, extinction of the infection possibly occurs when ${\mathcal{R}}_{0}^{s}<1<{\mathcal{R}}_{0}$ and the intensity of white noise is large. This would not happen in the deterministic models. The potential of using stochastic SIRC model for COVID-19 is to consider the environmental fluctuation that all affects the spread of the virus. The periodicity of outbreaks is possible due to the presence of time-delay (memory) in the transmission terms.

The authors believe that the stochastic SIRC model is an attempt to understand epidemiological characteristics of COVID-19. The model provides new insights into epidemiological situations when the environmental noise (perturbations) and cross-immunity are considered in the COVID-19 epidemic models. The combination of white noise and time-delay, in the epidemic model, has a considerable impact on the persistence and extinction of the infection and enriches the dynamics of the model. This work can be extended to include control variables for vaccination, treatment, and/or quarantine actions. A more sophisticated model is also required to investigate the dynamics of COVID-19 with immune system in cells level [42]. Fractional derivatives can also be included in the model to consider long-run memory [43, 44].

## Footnotes

1 Cross-immunity (or cross-reactivity) is a major evolutionary force that affects pathogen diversity (i.e. it drives viruses and microbes to be as distinct as possible from one another in order to avoid immunity detection, memory recognition, and clearance).
